# Training-Induced Neural Plasticity in Youth: A Systematic Review of Structural and Functional MRI Studies

**DOI:** 10.3389/fnhum.2020.497245

**Published:** 2021-01-18

**Authors:** Olga Tymofiyeva, Robert Gaschler

**Affiliations:** ^1^Department of Radiology & Biomedical Imaging, University of California, San Francisco, CA, United States; ^2^Department of Psychology, University of Hagen, Hagen, Germany

**Keywords:** brain, plasticity, training, adolescent, child, development, neuroimaging, MRI

## Abstract

Experience-dependent neural plasticity is high in the developing brain, presenting a unique window of opportunity for training. To optimize existing training programs and develop new interventions, it is important to understand what processes take place in the developing brain during training. Here, we systematically review MRI-based evidence of training-induced neural plasticity in children and adolescents. A total of 71 articles were included in the review. Significant changes in brain activation, structure, microstructure, and structural and functional connectivity were reported with different types of trainings in the majority (87%) of the studies. Significant correlation of performance improvement with neural changes was reported in 51% of the studies. Yet, only 48% of the studies had a control condition. Overall, the review supports the hypothesized neural changes with training while at the same time charting empirical and methodological desiderata for future research.

## Introduction

Training one's brain to improve one's abilities and well-being is an empowering idea. Doing so at a young age when brain plasticity is high may be especially pivotal (Kleim and Jones, 2008). In order to optimize existing training programs and develop new interventions for normally developing children and children with developmental disabilities, it is important to understand what processes take place in the brain during training. Moreover, training effects need to be put into the context of maturational processes. Neuroimaging, in particular magnetic resonance imaging (MRI), has enabled us to monitor training-induced effects in the brain non-invasively (Zatorre et al., 2012). Both structural and functional MRI methods can provide valuable insights into the underlying mechanisms of experience-dependent plasticity in adults as well as children, due to their non-invasive nature and sensitivity to brain tissue contrasts.

While there exist non-systematic reviews of neural changes with training in adults (Zatorre et al., 2012) and children (Jolles and Crone, 2012), no recent systematic reviews of training-induced neural plasticity in youth are available. This is an important gap, especially given the recent publication of new research studies on this topic, such as the neuroimaging study of training-induced plasticity in children with reading disability (Romeo et al., 2018).

The overall research aim of this systematic review was to investigate the extent of changes in MRI-derived parameters in youth with training and the quality of evidence for such change.

## Background and Hypotheses

The following sections will provide the theoretical and empirical background for training-related neuroplasticity, describe MRI methodology that can be used to map neural changes, and address developmental/maturational effects on training-related plasticity. In the last section, the research question and specific hypotheses will be presented.

### Neuroplasticity

Neuroplasticity has been defined as experience-driven change in neural structure and function (Rapoport and Gogtay, 2008; p. 181). The most striking examples of neuroplasticity are found in cases of trauma or invasive surgery, such as hemispherectomy—where one brain hemisphere is removed, often as a measure to stop seizures (Ismail et al., 2017). The degree to which brain functions linked to the removed hemisphere recover depends on multiple factors: e.g., the disorder that led to the surgery, the patient's age, and the capacity for plasticity of the affected brain regions (Ismail et al., 2017). The recovery can sometimes be remarkable, as in the case of a child who could speak again after the removal of the left hemisphere (Fenton et al., 2009).

Such dramatic examples aside, brain plasticity underlies everyday learning (Johnston, 2009). Summarizing neuroscience-based understanding of experience-dependent neural plasticity, Kleim and Jones (2008) highlight that while lack of use of brain functions can lead to degradation, training of a specific function can lead to enhancement of the specific function. Yet, training should be adequate in intensity and level of repetition, and over the course of time can lead to different variants of plasticity. While abundant animal work has contributed to the understanding of neuroplasticity, recently, it became possible to conduct human studies of training-induced plasticity using MRI methodology described in the following section.

### MRI Methodology

**Figure 1:**
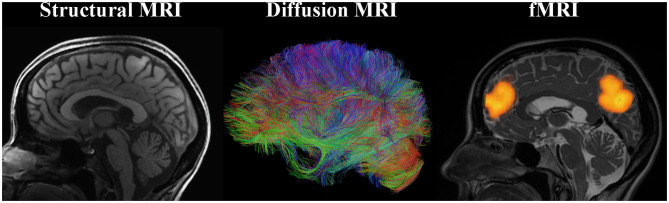
Main MRI modalities for measuring training-induced plasticity.

Several types of MRI allow for characterization of structure and function of the brain and can provide markers of neuroplasticity with training (Figure 1).

1. *Structural MRI*. Structural MRI (typically based on T1-weighted images) allows for delineating structures in the brain and assessing volume or thickness (Whitwell, 2009). In particular, with the voxel-based morphometry (VBM) approach one can automatically, semi-quantitatively analyze gray matter (GM) and white matter (WM) structure change over the course of training. The measures derived from the VBM analysis are often misleadingly described as GM “concentration” or “density.” However, such measures do not directly represent underlying neuronal densities. Even the GM “volume” derived using VBM depends on voxel intensities on T1-weighted images (where the boundary between WM and GM is drawn) and thus can be affected by any tissue properties that influence relaxation times (e.g., myelination).
2. *Diffusion MRI*. Diffusion MRI techniques have enabled assessment of WM microstructure and WM tracts (Mukherjee et al., 2008). For example, fractional anisotropy (FA) calculated based on the diffusion tensor imaging (DTI) quantifies the orientational dependence of water diffusion and reflects axonal diameter and density, myelination, etc. The principal eigenvector of the tensor in each voxel can provide information about the orientation of the WM fiber bundle passing through that voxel. By following these directional estimates, one can perform diffusion tractography and study structural connectivity in the brain. Diffusion MRI connectome analysis that utilizes graph theory has become a promising technique for investigating the human brain as a network of structural connections (Hagmann et al., 2010). Resolving crossing and kissing fibers and disentangling different contributions to unspecific metrics such as FA are the biggest challenges of diffusion MRI.
3. *Functional MRI (fMRI)*. Task fMRI assesses task-related variations in the blood oxygen-level-dependent (BOLD) signal in order to derive the intensity and spatial distribution of brain function (Logothetis et al., 2001). Neuronal activity leads to local increases in oxygen consumption and to the associated drop in oxygenated hemoglobin and increase in deoxy-hemoglobin. These changes in hemoglobin get rapidly overcompensated by increased blood flow to the active area of the brain. Because the two types of hemoglobin have different magnetic properties, MRI can measure the occurring changes. It is important to emphasize that upregulation of BOLD signal is not the same as upregulation of neuronal (electrical) firing. Rather, it is an indirect, vascular measure. Spontaneous brain activity measured using BOLD fMRI in the absence of a specific task can also be used to map the intrinsic, resting-state functional networks of the human brain (Kelly and Castellanos, 2014). While *functional connectivity* (FC) can be assessed during a task, a special place in research is dedicated to resting-state FC, measured when the study participant is not asked to perform any explicit task. These networks of synchronized spontaneous fluctuations consume a lot of energy and, remarkably, appear to contain the full repertoire of functional dynamics normally captured during various types of tasks (Smith et al., 2009; Laird et al., 2011). Although there is a robust relationship between functional and structural connectivities, they are far from equivalent (Honey et al., 2009). Finally, *effective connectivity* analysis using dynamic causal modeling (DCM) can help investigate neuronal dynamics of the brain network in that it assesses directionality of functional connections between brain regions.

Apart from the MRI modalities outlined above, many other MRI-based methods can provide additional information on brain function and structure. For example, magnetic resonance spectroscopic imaging (MRSI) quantifies metabolite concentrations not only in a single region of interest (single voxel), but spatially mapped over larger areas (Scheau et al., 2012). Spectroscopic imaging provides indices of neuronal viability [*N*-acetyl-aspartate (NAA)], brain energy metabolism (creatine), cell membrane integrity (choline), and glial cell population (myo-Inositol) among others (Scheau et al., 2012).

Any imaging technique used to compare local metrics over time and/or across individuals is susceptible to errors and biases introduced by the imaged volume selection (especially in MRSI), co-registration among images, and smoothing procedures, and these need to be taken into account.

### Training-Induced Plasticity Findings in Adults

The advent of advanced MRI techniques has enabled *in vivo* human studies of changes occurring in the brain when a person undergoes different types of training. A pioneering study showed that taxi drivers in London had larger posterior hippocampi compared with controls and that this difference was related to the length of the driving experience (Maguire et al., 2000). The most important limitation of this type of cross-sectional analysis is that one cannot differentiate the effects of training on the brain from pre-existing environmental and/or genetic differences. However, longitudinal studies with imaging being performed before and after the training intervention have also shown remarkable neuroplasticity effects in adults. In 2004, Draganski et al. (2004) used VBM to visualize brain changes in volunteers who have learned to perform a three-ball juggling task. They showed a transient increase in GM regions associated with motion processing that was linked to the juggling performance (Draganski et al., 2004). Numerous MRI studies of training-induced neuroplasticity in adult subjects have followed (including an experimental equivalent of the taxi driver study (Lövdén et al., 2012) and were reviewed extensively (Draganski and May, 2008; Zatorre et al., 2012). Different types of trainings were studied, ranging from juggling, to learning math, to meditating. Even a mere “action observation training” in healthy adults promoted competence and modified GM structure compared with controls who watched videos of landscapes (Rocca et al., 2017). In addition to structural and microstructural changes, metabolic and fMRI-derived changes were observed with training (e.g., juggling, Sampaio-Baptista et al., 2015). This *in vivo* evidence demonstrated functional and structural reorganization of the human brain. Yet, questions about the underlying biological mechanisms of the observed changes remained largely unanswered.

### Training-Induced Biological Changes Measured With MRI

While numerous biological changes can affect MRI measures (Zatorre et al., 2012), only some of them are sufficiently backed up as candidate mechanisms by animal training studies that used “rigorous experimental design and a combination of high spatial resolution MRI and immunohistochemistry” (Thomas and Baker, 2013; p. 9). Two animal studies providing this type of evidence are summarized below, and an overview of biological changes can be found in Table 1.

**Table 1:** Training-induced biological changes that affect MRI measures.

**Training-induced biological change**	**Schematic representation of the biological change**	**MRI changes**	**Developmental considerations**
**TRAINING-INDUCED CELLULAR MECHANISMS SHOWN TO MODULATE MRI MEASURES**			
Changes in synapses and remodeling of neuronal processes (dendritic branching)	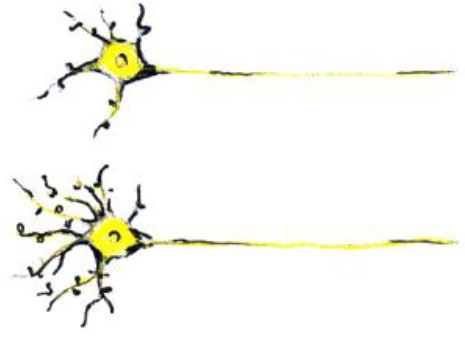	- Can cause apparent increase in GM thickness/volume - Can cause changes in fMRI activation and connectivity	- Synaptic pruning and GM thinning in adolescence (Giedd et al., 1999)
Changes in astrocytes	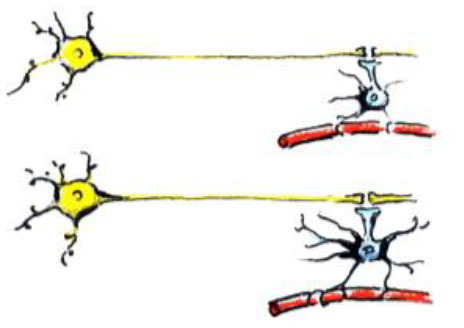	- Can cause apparent increase in GM and WM thickness/volume - Can cause changes in fMRI activation and connectivity	- Increases in astrocytic complexity and process length (GM) as primates mature (Robillard et al., 2016)
Changes in myelination	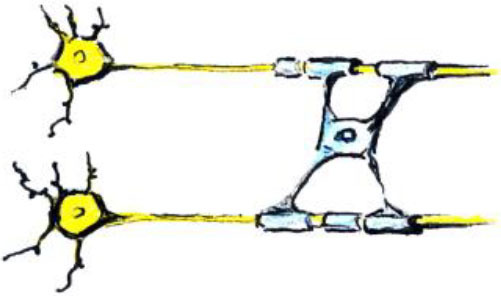	- Can cause changes in diffusion MRI metrics - Can cause increase in WM thickness/volume - Can cause changes in fMRI activation and connectivity - Can change WM/GM boundary on T1 and T2 images and thus apparent GM volume	- Myelin increases in childhood and adolescence (Lebel and Beaulieu, 2011)
Change in density of capillaries	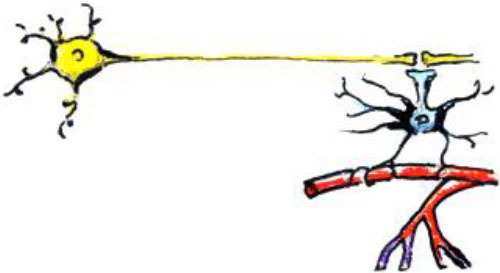	- Can cause changes in diffusion MRI metrics - Can cause apparent increase in WM thickness/volume - Can cause changes in fMRI activation and connectivity	- Vessel density changes with age (Miyawaki et al., 1998)
**TRAINING-INDUCED PHYSIOLOGICAL, BEHAVIORAL, COGNITIVE, AND MOOD CHANGES THAT CAN MODULATE MRI MEASURES**			
Changes in mood, mental activity at rest, and strategies during task	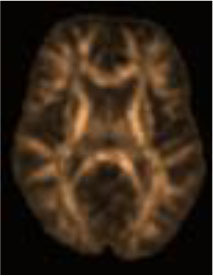	- Can cause changes in fMRI activation and connectivity	- Emotion regulation changes with age (Theurel and Gentaz, 2018)
Changes in head motion, breathing, and cardiac pulsation	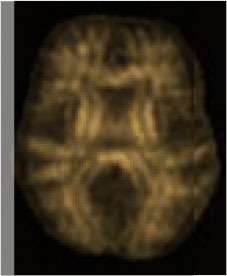	- Can cause artifactual changes in structural, diffusion MRI, and fMRI metrics	- Head-movement likelihood changes with age (Satterthwaite et al., 2012)

See Zatorre et al. (2012) for a similarly structured overview of structural changes.

*GM, gray matter; WM, white matter*.

In one study, Lerch et al. (2011) tested if different types of training (spatial in comparison with non-spatial) can cause structural brain changes in mice. Animals trained on the spatial version of the water maze had hippocampal volume increases, whereas those trained on a non-spatial cued version of the maze displayed striatal increases. Importantly, these MRI-derived volume changes correlated with axonal growth-associated protein-43 (GAP-43) staining and not with markers of neuron or astrocyte number or size. This indicates that the measured morphological changes are due to *remodeling of neuronal processes* and not due to neurogenesis.

In another study, Blumenfeld-Katzir et al. (2011) examined DTI correlates of spatial learning in a group of rats. The authors found training-related changes of DTI indices in the dentate gyrus, cingulate cortex, piriform cortex, sensory cortex, and corpus callosum. The histological analyses showed that increases in *synapses* and *astrocytes* may have contributed to the changes in the diffusion indices in the dentate gyrus. At the same time, an increase in *oligodendrocytes* forming the *myelin* sheaths was identified as the mechanism linked to the increase in FA in the corpus callosum.

In summary, the findings from these two animal studies suggest that the observed MRI changes in human training studies may be due to changes in axonal growth and myelination, as well as in change in synapses and astrocytes. Although previous publications referred to continuing neurogenesis as a candidate mechanism underlying visible MRI changes with training (Zatorre et al., 2012), this is highly unlikely, given the rapid decline in the number of new neurons emerging after birth in the human brain (Sorrells et al., 2018).

Another animal study sheds light on one more important source of training-induced changes in MRI metrics, both structural and functional. Morland et al. (2017) demonstrated an increase in *density of capillaries* in the sensorimotor cortex, and in the dentate gyrus of hippocampus of wild-type mice after 7 weeks of exercise, compared with sedentary controls.

All of the biological processes described above—changes in synapses, axons and dendrites, astrocytes, myelination, and density of capillaries—can affect metrics derived from different types of MRI, including fMRI (Table 1). Changes in fMRI signal with training, however, can happen faster, before any MRI-detectable structural or microstructural changes take place. An organizing framework for interpreting the plasticity of task-evoked functional activity has been proposed (Kelly and Garavan, 2005). According to this framework, the way fMRI signal will change with training will depend on a number of factors:

1. The changes in cognitive processes involved in task performance. For example, shift in strategy used by the subject to accomplish a task can lead to a *reorganization* of task-evoked activity. Similarly, a *redistribution* of the fMRI signal is expected when practicing the task increases reliance on task-specific brain regions and decreases reliance on regions responsible for control and attentional processes (such as the prefrontal cortex and anterior cingulate cortex).
2. Task domain. Practice has divergent effects on functional activations in different brain regions, depending on their functional domain: e.g., motor and sensory tasks vs. higher-level cognitive tasks.
3. The timepoint at which imaging is performed. Ideally, one would image the entire trajectory of practice-related brain changes, in order to make strong conclusions with respect to the plasticity mechanisms.

Kelly and Castellanos (2014) adapted this framework to the interpretation of the effects of practice on intrinsic FC. To use training of a sensorimotor task as an example, the task would cause a redistribution of intrinsic FC. Soon after the subject starts the practice, intrinsic FC between task-domain regions and higher-level cognitive and associative cortices will increase. As the subject continues the practice and his or her performance plateaus, connectivity with the higher-level cognitive and associative cortices goes back to baseline, whereas connectivity within the network of task-domain regions increases.

As indicated in the previous paragraphs, one should keep in mind that the specific biological changes underlying the changes in MRI measures will depend on the type of training, as well as on its duration. Learning a new skill or strategy is a multistage process, with the fast learning stage and the subsequent slow learning engaging different brain regions and different cellular mechanisms (Dayan and Cohen, 2011).

Finally, training-induced physiological and behavioral changes can modulate MRI measures without directly reflecting neural neuroplasticity. For example, emotion regulation training can help subjects keep the head still during the scan and thus reduce motion artifacts. Similarly, cardiac pulsation changes after training can lead to measurable artifactual changes in fractional anisotropy in different areas of the brain (Pierpaoli et al., 2003).

### Plasticity in the Developing Brain and Methodological Considerations

The young brain can display levels of neuroplasticity that significantly exceed those of the adult brain (Kleim and Jones, 2008). Here, we specifically focus on *training-induced, experience-dependent neuroplasticity*. This can be differentiated from *developmental neuroplasticity*, which reflects genetically encoded, time-dependent, sequenced maturational processes (Ismail et al., 2017). While the most drastic genetically encoded changes happen perinatally, the maturational processes continue throughout adolescence (Kadosh et al., 2013), both in gray matter (Giedd et al., 1999), and white matter (Lebel and Beaulieu, 2011). Training-induced plasticity, as one variant of experience-dependent plasticity, is especially abundant in the developing brain (Ismail et al., 2017). This adaptation to the environment is subserved by changes in neuronal processes (axons and dendrites), synapse formation and elimination, and myelin remodeling, as discussed in the previous section.

When conducting MRI studies of training-induced changes in children and adolescents, it has to be taken into account that developmental and experience-dependent neuroplasticity might occur simultaneously (Laube et al., 2020). Table 1 lists side-by-side examples of training-induced neuroplasticity and the overlapping developmental plasticity that might be considered. For example, due to maturational processes, cortical thickness exhibits a preadolescent increase followed by a thinning in adolescence and young adulthood (Giedd et al., 1999). In addition, there are regional differences in maturation with primary motor and sensory areas developing sooner than higher level association and multimodal areas (Gogtay et al., 2004). White matter volume, on the other hand, steadily increases up to adulthood (Gogtay et al., 2004). Cortical thickening in childhood may reflect changes in the level of synaptogenesis. The dissimilar pattern in gray matter and white matter in adolescence is likely to reflect pruning of redundant or unused synaptic connections (loss of gray matter) and the strengthening of relevant connections *via* myelination based on environmental input and experience (white matter volume increase) (Huttenlocher, 1990). Microstructural maturation of individual white matter tracts also demonstrates dependence on age and location, affecting diffusion MRI metrics (Lebel and Beaulieu, 2011). A longitudinal resting state fMRI (rs-fMRI) study demonstrated, on one hand, established primary visual and motor-sensory networks with minimal intersubject variability in neonates, and on the other hand, age- and sex-dependent maturation and more intersubject variability in higher-order association networks in the first 2 years of life (Gao et al., 2015).

The complex changes in MRI-derived metrics observed with age need to be taken into account when interpreting brain-behavior correlates. Opposite correlations with performance can be observed in children compared with adults. For example, Schnack et al. (2015) analyzed cortical thickness in relation to intelligence in a large longitudinal sample of over 1,000 MRI scans in subjects of age between 9 and 60 years. The researchers showed that at younger age, thinning of the cortex was associated with higher intelligence; however, this relationship was reversed in young adults, where higher IQ correlated positively with increase in cortical thickness (Schnack et al., 2015). Accordingly, researchers discuss how volume growth in specific areas induced by training or other environmental challenges is backed up by selection processes leading to a return to baseline in overall volume (Wenger et al., 2017).

A special concern for training studies also stems from the risk of artifactual findings due to age-related differences in parameters that do not directly reflect neural mechanisms, like head motion or physiological parameters such as neurovascular coupling (Zuo et al., 2017). Because of the ongoing maturational processes in the brain and changes in physiology and behavior during scanning with age, study design considerations are especially critical for MRI studies of training-induced changes in children and adolescents. In their review of studies investigating training-induced structural plasticity in humans, Thomas and Baker (2013) emphasized the importance of a control group.

Ideally, a *randomized-controlled study design* is employed, in which participants are randomized to a training or control (active or waiting) group (Table 2). With this study design, MRI measures that display a significant interaction between group and timepoint (before vs. after the training) provide evidence for training-dependent plasticity. The selection of an appropriate control group is one of the biggest challenges in training research (Green et al., 2014). Active control groups are usually considered to be the best choice, because passive control groups fail to rule out numerous possible confounds. If clinical populations are studied, the control group should have the same disorder as the training group. Sometimes it is not possible due to ethical considerations (e.g., withholding treatment from depressed adolescents). Additional inclusion of healthy age-matched controls (also randomized to training or waiting) would be ideal to see the differences between the training-induced effects in patients and healthy controls but would require considerable additional recourses.

**Table 2:** Three main types of study designs used in MRI studies of training-induced plasticity.

**Study design**	**Schematic representation of the study design**	**Advantages/drawbacks**
Randomized-controlled study design	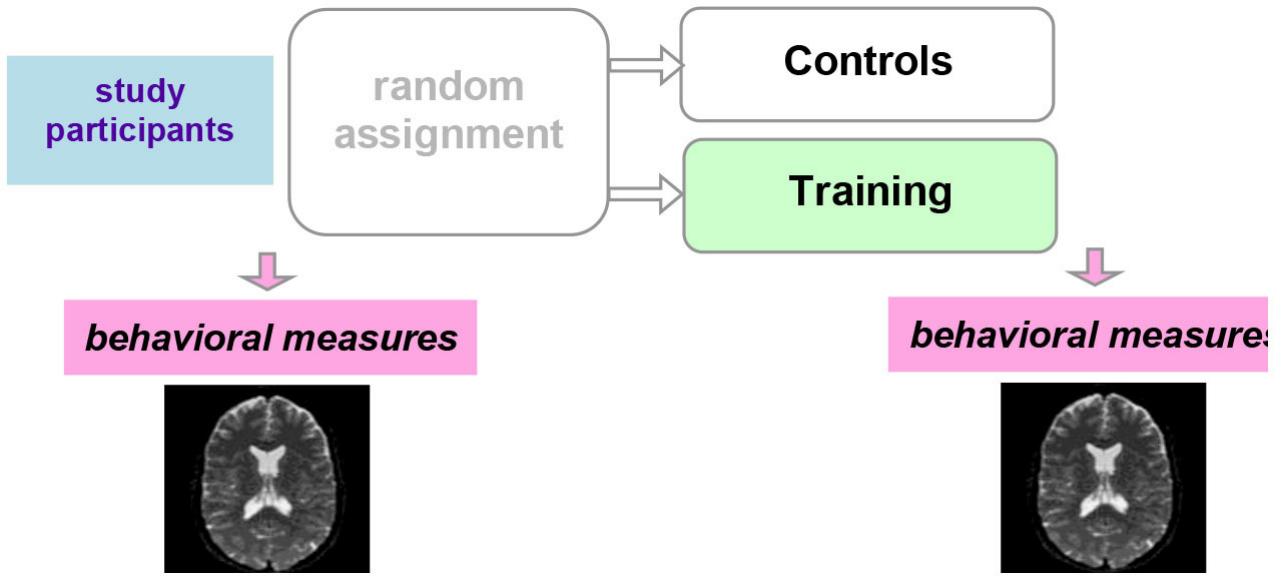	The “gold standard”: best control of variables
Controlled within-subject study design	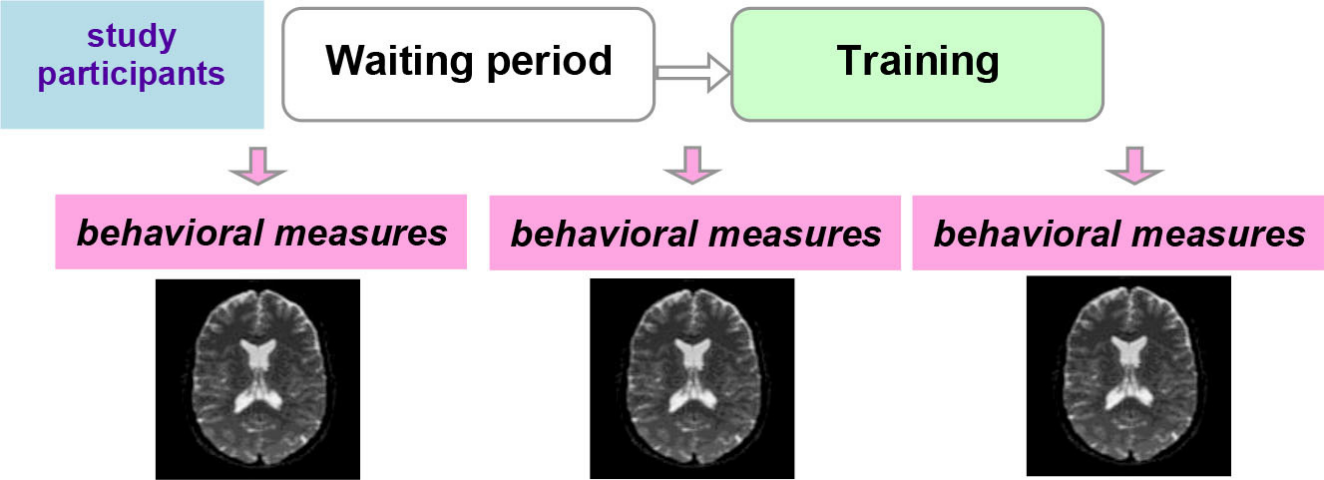	Avoids the effect of between-subjects variance, however, can introduce seasonal effects and should be counterbalanced
Within-subject study design	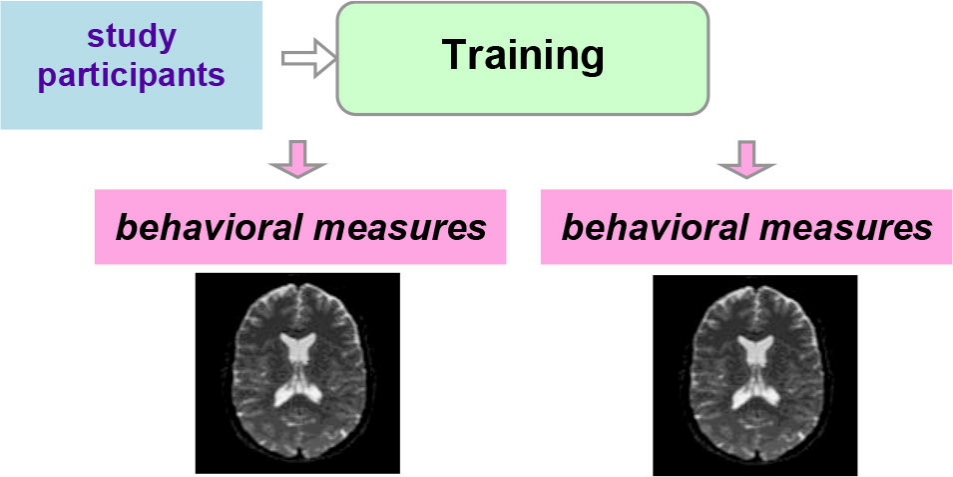	No control of variables; allows for studying neural correlates of behavioral changes

Additional strength of evidence can be provided by correlation of observed MRI changes with behavioral changes in the training group. If detected changes in MRI measures are the true result of training, it is reasonable to expect that the change in MRI measures would correlate with changes in training-related behavioral measures. Moreover, dose-dependence of the training effects can be studied by comparing, for instance, one low-dose and one high-dose group with the same training.

Another possibility is a *controlled within-subject design*. In this study design, subjects are scanned three times. No training takes place between the first two scans, and this time interval serves as a control condition. The training then takes place between the second and the third scans (Table 2). This study design can be potentially more powerful than a between-subjects design, as it avoids the effect of between-subjects variance (Poldrack, 2000). One potential problem is the seasonal effects that can differently affect study participants during the no-training and training periods (e.g., school time vs. summer vacation). A counterbalanced version of this study design solves the problem of potential seasonal effects but is not always possible (e.g., when participants acquire skills during training that they are encouraged to use regularly in the everyday life after the training is completed). One can, as a compromise, shift the timelines of different participants (starting points) across different seasons.

Finally, a simple *within-subject design* can be employed, which would be considered to provide the weakest evidence. As mentioned above, especially in the context of the developing brain, changes detected pre-/posttraining can simply reflect maturational processes. A methodological possibility within this option is to analyze correlation of neural changes with changes in behavior or compare “responders” with “non-responders”.

### Research Question and Hypotheses

Given the aforementioned availability of neuroimaging techniques suitable for studying training-induced plasticity in children and adolescents, one would expect that such studies would emerge and potentially demonstrate measurable brain changes. There are, however, no systematic reviews available for this type of research literature. In this systematic review, we aimed at closing this gap and answering the following research question: Are there measurable neural effects of training in children and adolescents that can be captured by MRI? Here, we define *training* as the process of improving cognitive function or behavior by means of practice. Our review is thus focused on training-induced neuroplasticity, which is a type of experience-dependent neuroplasticity that follows an intentional repeated activity (namely, training). Based on previous literature, including the reviews of relevant studies in children and adults (Jolles and Crone, 2012; Zatorre et al., 2012), the following hypotheses were formulated:

*Hypothesis 1 (H1)*: There are differences in the MRI-derived neural metrics before and after the training in children and adolescents.
*Hypothesis 2 (H2)*: There are differences in changes of the MRI-derived neural metrics between the training group and control group in children and adolescents.
*Hypothesis 3 (H3)*: The improvement in performance in the training group (or among responders) correlates with the MRI-derived neural changes in children and adolescents.

More generally, by performing this systematic review, we also aimed to obtain the data about the number of published research studies on training-induced neuroplasticity in youth, as well as about the utilized MRI techniques, study designs, populations, and types of training.

## Methods

Open Science Framework (OSF) preregistration of the project was conducted on 7 May 2018 (https://osf.io/ajr9k). The preanalysis document describes the hypotheses stated above and the methodology as follows.

To review the research literature on training-induced neuroplasticity in children and adolescents, electronic database search and data synthesis were conducted as described in the following sections.

### Search Strategy

Electronic databases (PubMed, PsycINFO, and Google Scholar) were searched for all MRI studies of training-induced neural changes in youth published to date. The search was performed in June 2018 and updated in August 2020. To find relevant abstracts, the neuroplasticity-related search terms were *neural, brain, (neuro)plasticity, change, reorganization*, and *reorganisation*. The training-related search terms were *trainingitherapy, intervention*, and *exercise*. The imaging-related search terms were *(f)MRI, magnetic resonance*, and *DTI*. The population-related search terms were *children, adolescents*, and *youth*.

The following *exclusion criteria* were used:

- Studies that are not in English
- Cross-sectional studies (studies that do not include MRI-based measurements before and after the training)
- Animal studies
- Studies with participants older than 18 years
- Non-MRI studies (studies utilizing other types of neuroimaging)
- Studies involving training/therapy/intervention that is not of cognitive or behavioral nature
- Studies that are not peer reviewed.

**Figure 2:**
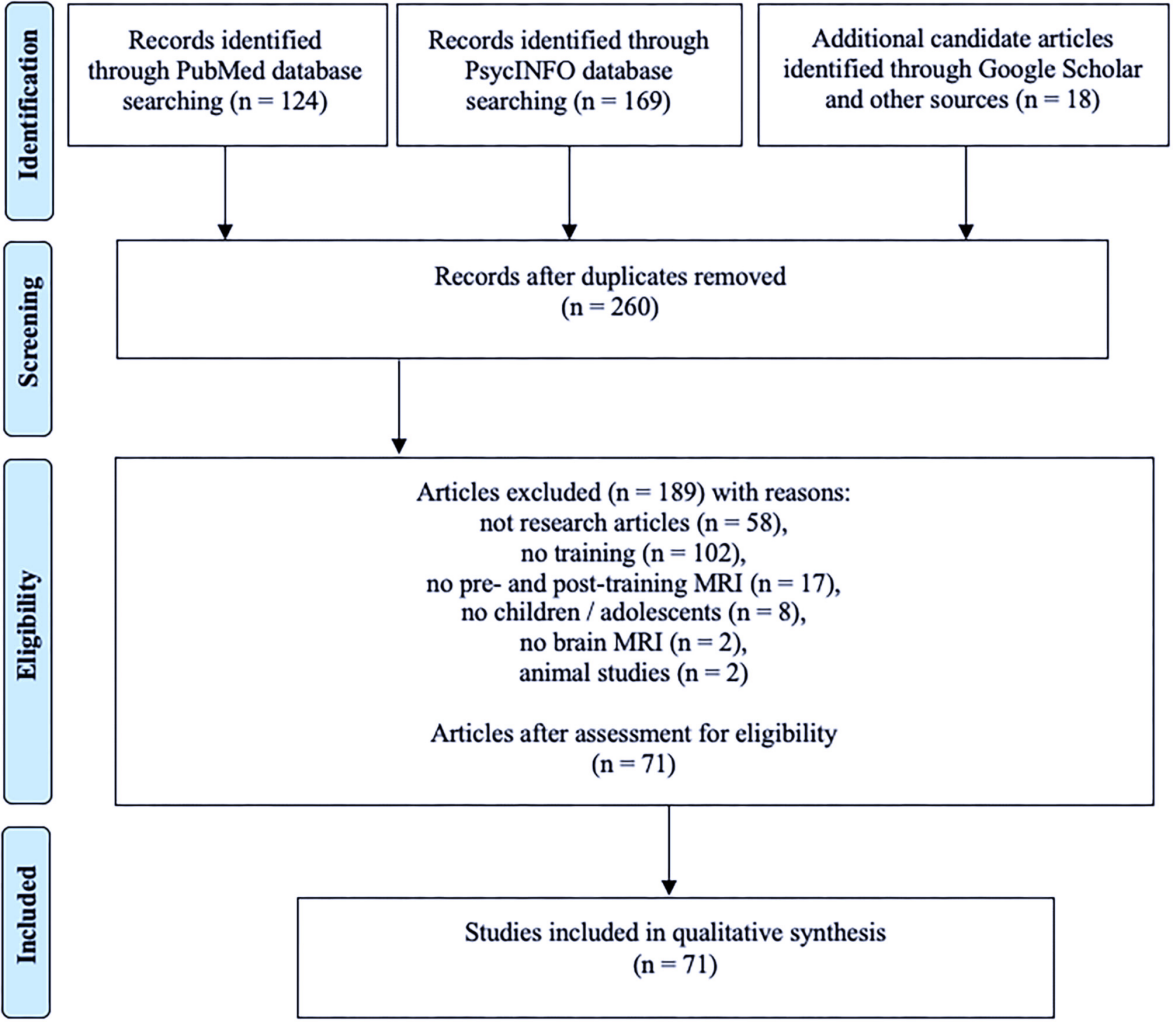
Literature search flow using PRISMA guidelines (Moher et al., 2009).

The process of the systematic search is summarized in Figure 2. PubMed and PsychINFO search initially resulted in 232 non-overlapping records. Eligibility analysis resulted in exclusion of 189 records with the following reasons: 58 of the records were not research articles (review articles, dissertations, book chapters, etc.), 102 articles did not contain training as part of the study protocol, 17 articles did not contain pre- and posttraining MRI (e.g., prognostic pretraining MRI only), in eight of the studies the participants were adults, two of the studies did not contain brain MRI, and two articles described animal studies. Additional 18 articles meeting the eligibility criteria were identified through Google Scholar search and other sources (such as screening the references in other articles). The updated search in August 2020 resulted in 10 additional articles. The total number of articles included in qualitative synthesis was 71.

### Data Synthesis

A high heterogeneity was expected among the studies with respect to the study population, type and duration of training, studied brain regions and, importantly, the specific MRI method. Different MRI methods capture different biological phenomena (e.g., changes in white matter myelination, cortical thickness, functional connectivity), and they most likely have different sensitivity to changes and different dynamics (e.g., fMRI changes may be noticeable with shorter trainings). Therefore, a *narrative synthesis* was chosen instead of a meta-analysis, as meta-analysis is not recommended for diverse study types (Centre for Reviews Dissemination, 2009). The narrative synthesis framework proposed by Popay et al. (2006) can be utilized for this type of analysis. As main elements, the framework suggests to (1) develop a theory of how the intervention works and for whom, (2) preliminarily synthesize the findings of the included studies, (3) further explore relationships in the data, and (4) assess the robustness of the synthesis (Popay et al., 2006; p. 11). These four elements were approached in an iterative manner using the tools proposed by Popay et al. (2006). For the purposes of this review article, the first element of the framework was interpreted in terms of neural changes with training (H1) or neural changes with training compared with a control condition (H2) as a measure for whether the intervention works. Our hypothesis (H3) refers to the third element of the framework (further explore relationships in the data) and states that the improvement in performance in the training group (or among responders) correlates with the MRI-derived neural changes in children and adolescents.

## Results

Table 3 provides a brief summary of the study design, population, training type, MRI methodology, and results for each of the 71 included studies. Before we report on the hypotheses, we characterize the type of studies targeting training-induced neuroplasticity in children and adolescents. This is relevant for identifying for which combination of training, target group, and MRI measure we have a reasonable number of studies and which approach is less represented in the literature.

**Table 3:** Summary of the included articles.

**First author (year)**	**Study design**	**Sample size**	**Age (mean or range) and population**	**Training**	**Type of MRI**	**Results: (1) neural changes; (2) comparison with controls; (3) correlation with performance**
**NORMAL POPULATION, ADOLESCENTS**						
Kleibeuker et al. (2017)	Random. contr. (active contr.)	16 + 16	15.8 and 16.2 years old	(c) Divergent thinking training and active controls: rule switching training; 2 weeks, 8 sessions	Task fMRI; alternative uses task	(1) Increased activation in L middle temporal/angular gyrus/occipital lobe; (2) Group × timepoint interaction at trend level; (3) change in activity in L middle lateral PFC correlated with change in alternative uses fluency
Yuan et al. (2020)	Contr. within-subject	38	16.5 years old	(c) Mindfulness meditation-based Training for Awareness, Resilience, and Action (TARA); 12 wks	sMRI	(1) Decreased GM volume in the L posterior insula and to a lesser extent in the L thalamus and L putamen, not in controls; (2) –; (3) –
Patsenko et al. (2019)	Random. contr. (active contr.)	47 + 48 controls	12.8 years old	(c) Meditation-based video game, tenacity; 2 weeks	rs-fMRI (L dlPFC-seeded), DTI	(1) Increase in L dlPFC-L IPC rs-FC (not corrected); (2) significant group effect in the change in rs FC between L dlPFC and L IPC, not for FA; (3) changes in L dlPFC – IPC rs FC and changes in FA of the corresponding tract correlated with attentional improvement
**NORMAL POPULATION, CHILDREN**						
Ekerdt et al. (2020)	Random. contr. (passive and active contr.)	20 + 19 active controls + 20 passive controls	4 years old	(c) 3 weeks of word learning	DTI	(1) –; (2) FA increase in the left precentral WM; (3) –
Wang et al. (2017)	Random. contr. (passive contr.)	26 + 25	6.9 and 7 years old	(c) Abacus-based mental calculation (AMC) training; 2 h/week over 5 school years	Task fMRI; executive function task	(1) –; (2) group × timepoint interaction in R intraparietal sulcus/lobule and R superior frontal gyrus/supplementary motor area; (3) –
Newman et al. (2016)	Non-random. contr. (act. contr.)	14 + 14	8.3 and 8.2 years old	(c) Block play training; 5 sessions; act. controls: word/spelling board game	Task fMRI; mental rotation task	(1) Increased activation in anterior lobe of the cerebellum extending into R parahippocampus and fusiform gyrus, not in controls; (2) –; (3) –
Jolles et al. (2016b)	Uncontr.	18	7.7–8.1 years old	(c) Intense math tutoring; 8 weeks	DTI and tractography	(1) No FA changes; (2) –; (3) FA changes in L superior longitudinal fasciculus linking frontal and temporal cortices correlated with behavioral gains
Supekar et al. (2015)	Uncontr.	28	8.6 years old	(c) Intensive cognitive/math tutoring; 8 weeks	Task fMRI; arithmetic problem-solving task	(1) decreased/normalized amygdala activity and effective connectivity of amygdala in high math-anxiety children; (2) –; (3) changes in amygdala activity correlated with reductions in math anxiety
Rosenberg-Lee et al. (2018)	Non-random. contr. (passive contr.)	19 + 15	8.5 and 8.8 years old	(c) Math training; 8 weeks	Task fMRI; arithmetic task	(1) Increased activity in L anterior hippocampus, not in controls; (2) significant compared with controls; (3) gains in memory-based strategies correlated with decreased lateral fronto-parietal activity and increased hippocampus-parietal FC
Brehmer et al. (2016)	Random. contr. (passive contr.)	16 + 12 controls	11.0 and 11.2 years old	(c) Mnemonic skill training; 4 strategy instruction and practice sessions	Task fMRI; memorizing word pairs	(1) Decreases in subsequent-memory (SM) effects in several brain areas; (2) changes in SM effects in L frontal regions compared with controls; (3) –
Jolles et al. (2016a)	Non-random. contr. (passive contr.)	21 + 21	8.6 and 9 years old	(c) One-on-one math tutoring; 22 lessons over 8 weeks	rs-fMRI; FC	(1) Increased FC of intraparietal sulcus (IPS) with lateral prefrontal cortex, ventral temporal-occipital cortex, and hippocampus, not in controls; (2) –; (3) increased IPS FC correlated with performance gains
Jolles et al. (2013)	Uncontr.	9	12.2 years old	(c) Working memory task; 6 weeks	rs-fMRI; FC	(1) No practice effects found in children; (2) –; (3) –
Jolles et al. (2012)	Non-random. contr. (passive contr.)	10 + 6	12.4 and 12.7 years old before excl.	(c) Working memory task; 6 weeks	Task fMRI; working memory task	(1) Nonsignificant; (2) –; (3) –
Park et al. (2015)	Uncontr.	29	8.2 years old	Arts training (creative movement and musical arts); 15 weeks	sMRI, DTI	(1) Increased cortical thickness in L postcentral gyrus and superior parietal lobule, decreased mean diffusivity in R posterior corona radiata and superior longitudinal fasciculus; (2) –; (3) cortical thickness changes correlated with changes in cognitive measurements
Bauer et al. (2019)	Random. contr. (active contr.)	40	11.8 years old	(c) Mindfulness; 8 weeks, 5 days/week	Task (emotional faces) and rs-fMRI; FC	(1) –; (2) reduced R amygdala activation to fearful faces, stronger FC between R amygdala and ventromedial PFC during the viewing of fearful faces; (3) lower stress associated with reduced R amygdala activation to fearful faces
Delalande et al. (2020)	Random. contr. (active contr.)	64 children + 59 adolescents	10 and 16 years old	(c) Inhibitory control (IC) training 15 min/day, 5 days a week for 5 weeks (25 sessions) on a tactile tablet at home	sMRI	(1) –; (2) not stat. sign. changes in cortical thickness and cortical surface area in several PFC subregions (e.g., the pars opercularis, triangularis, and orbitalis of the inferior frontal gyri); (3) changes in pars orbitalis and L pars triangularis, R pars opercularis correlated with IC efficiency changes (exploratory)
**SPECIAL POPULATION, ADOLESCENTS**						
Stevens et al. (2016)	Uncontr.	18	15.2 years old adolescents with ADHD	(c) Working memory training (Cogmed™); 25 sessions over 5 weeks	Task fMRI; nonverbal Sternberg WM task	(1) Increased activity in several frontal, parietal, and temporal lobe regions; (2) –; (3) activation gains correlated with cognitive and clinical changes
Choi et al. (2015)	Random. contr. (active contr.)	13 + 17 active controls	15.8 and 16.0 years old adolescents with ADHD	Methylphenidate + 6-week exercise; active controls: methylphenidate + 6-week education	Task fMRI; Wisconsin card sorting test	(1) Increased activity in R frontal and L parietal lobe, decreased activity of R temporal lobe; (2) increased activity in R frontal lobe compared to controls; (3) change in activity in R prefrontal cortex in all teens neg. correlated with change in symptoms
Strawn et al. (2016)	Uncontr.	9	13 years old youth with generalized, social, and/or separation anxiety disorder and a parent with bipolar disorder	(c) Mindfulness-based cognitive therapy for children (MBCT-C); 12 weeks	Task fMRI; continuous processing task with emotional and neutral distractors (CPT-END)	(1) Increased activation in insula, lentiform nucleus, thalamus, L ACC; (2) –; (3) changes in insula and ACC correlated with decreases in anxiety
Maslowsky et al. (2010)	Nonrandom. contr. (healthy passive contr.)	7 + 10 healthy controls	13.4 and 14.5 years old youth with generalized anxiety disorder (GAD)	(c) CBT	Task fMRI; emotional faces	(1) –; (2) increased R VLPFC and amygdala activation to angry faces, relative to controls; (3) no correlation
Kim et al. (2018)	Uncontr.	23	14 years old before excl. teens, bullies	(c) CBT for externalizing behavior problems; 8 sessions	rs-fMRI	(1) Decreased low-frequency fluctuations in inferior parietal lobule, lingual, interior frontal, and middle occipital gyri; (2) –; (3) changes in inferior frontal gyrus correlated with changes in externalizing behavior scores (uncorrected)
Stoddard et al. (2016)	Uncontr.	10	14.1 years old before excl. youth with disruptive mood dysregulation disorder	(c) Interpretation bias training; 4 sessions of daily training	Task fMRI; face-emotion processing task	(1) Increased neural activation to subtle expressions of happiness relative to subtle expressions of anger in lateral OFC; (2) –; (3) –
Chattopadhyay et al. (2017)	Nonrandom. contr. (healthy passive contr.)	17 + 30 healthy controls	15.4 and 15.6 years old adolescents with MDD	(c) CBT; appx. 24 weeks	rs-fMRI; FC	(1) Increase in rsFC between R insula and L ACC, with baseline case-control differences reduced; (2) between-group effect at baseline differed from between-group difference after T; (3) FC changes correlated with changes in depression severity
Chuang et al. (2016)	Non-random. contr. (healthy passive contr.)	13 + 20 healthy controls	15.8 and 15.6 years old female adolescents with MDD	(c) CBT; appx. 30 weeks	Task fMRI; affective go/no-go task	(1) Decreased (normalized) activation in response to happy in OFC; (2) group × timepoint interaction in OFC; (3) nonsignif. corr. with improvements in symptoms
Straub et al. (2015)	Nonrandom. contr. (passive contr.)	10 + 12	16.4 and 16.5 years old medication naïve adolescents with MDD	(c) Cognitive behavioral group therapy (CBT-G); 5 weeks	Task fMRI; monetary reward task	(1) Decreased activation in amygdala, hippocampus, and ACC, not in controls; (2) group × timepoint interaction in L amygdala, L hippocampus and R ACC; (3) changes in ACC correlated with symptom improvement
Karoly et al. (2019)	Random. contr. (active contr.)	19 + 18	18.8 years old before excl. cannabis-using youth	Cannabis Approach Avoidance Training (CAAT); 3 weeks, 2 times/week	Task fMRI; visual cannabis cue-reactivity task	(1) Nonsign. small-to-medium decreases in amygdala and medial PFC activation to cannabis cues; (2) not sign.; (3) –
Alves-Pinto et al. (2015)	Random. contr. (passive contr.)	10 + 6 controls	12.8 and 16.4 years old youth with neuromotor impairments	Individualized piano lessons; 18 months; 30–45 min 2× week	Task fMRI; finger tapping	(1) Increase in endogenous connectivity from L primary motor cortex to R cerebellum with T.; (2) group × timepoint interaction for this change, none for BOLD activation during task, task-depend. connectivity or driving input; (3) –
Catharine et al. (2019)	Uncontr. (comparing with control brain regions)	16	15-year 8-month adolescents with traumatic brain injury (TBI)	(c) Cognitive computerized training program; 8 weeks, 5 times/week, 40 min/session	sMRI	(1) Not sign.; (2) GM volume decrease of 0.5% in executive functioning regions compared with control regions, between immediately postintervention and 6 months follow-up, compared with control brain regions; (3) negative correlation between change on the Digit Symbol Substitution test and change in volume of putamen (explorat)
O'Neill et al. (2012)	Uncontr.	5	13.5 years old youth with OCD	(c) Exposure-based CBT; 12 weeks	MRSI	(1) Decrease in *N*-acetyl-aspartate (NAA) and creatine in L pregenual ACC and increase in choline (Cho) in R thalamus; (2) –; (3) Cho increase in L thalamus correlated with symptom decrease
Han et al. (2012)	Uncontr.	14	14.2 years old before excl. adolescents from dysfunctional families with online game addiction	Family therapy intervention; 3 weeks, 5 sessions + cohesion interactions 4 days/week for >1 h	Task fMRI; images of parental affection and scenes from online games	(1) Increase of activation of caudate nucleus in response to affection cues, decrease of activation in L middle frontal gyrus in response to game stimulation; (2) –; (3) increase in caudate correlated with improvement in family cohesion and negatively with changes in online game playing time
Garrett et al. (2019)	Non-random. contr. (healthy passive contr.)	20 + 20	9–17 years old adolescents with PTSD	(c) Trauma-focused CBT	Task fMRI; facial expression task	(1) –; (2) –; (3) improvement in symptoms of PTSD correlated with decreasing activation in posterior cingulate, mid-cingulate, and hippocampus; improvement in dissociative symptoms correlated with decreasing activation in the amygdala
Cisler et al. (2016)	Uncontr.	20	13.9 years old before excl. adolescent girls with pediatric PTSD	(c) Trauma-focused CBT; 12 weeks, 60–90 min weekly sessions	Task fMRI; amygdala FC during cognitive reappraisal	(1) –; (2) –; (3) decreased FC between amygdala and insula while re-appraising correlated with symptom improvement
Mata et al. (2016)	Uncontr.	16	13.9 years old adolescents with excess weight	Weight loss intervention (CBT + physical activity + diet counseling); 12 weeks	Task fMRI; risky-gains task	(1) Reduced activation in midbrain, striatum, hippocampus, superior temporal gyrus, and lateral OFC; (2) –; (3) changes in insula activation correlated with reduction in body mass index (BMI) and fat percentage
Bernardoni et al. (2016)	Uncontr.	35	15.5 years old adolescent/young adult female patients with acute anorexia nervosa	Standardized weight restoration therapy; ~3 months	sMRI: cortical thickness and volumes of select subcortical regions	(1) Increased (normalized) cortical thickness; (2) –; (3) no correlation with psych. symptom improvement, only with weight restoration
Singh et al. (2020)	Random. contr. (active contr.)	34	14 years old symptomatic youth at high risk (HR) for bipolar disorder	Family focused therapy for high-risk youth (FFT-HR)	rs-fMRI	(1) Increased VLPFC-anterior DMN FC, not in controls; (2) sign. treatment by time interaction; (3) reduction in depression inversely correlated with enhanced anterior DMN connectivity
**SPECIAL POPULATION, CHILDREN**						
de Oliveira Rosa et al. (2019)	Random. contr. (active contr.)	10 + 10 active controls	6–13 years old children with ADHD, medicated with stimulants	(c) Computerized cognitive training program (ACTIVATE™)	Task fMRI; working memory (wm) N-back task, sustained attention task, inhibitory control Go-NoGo task	(1) –; (2) group × time × wm-load interaction effect in R insula and putamen and L thalamus and pallidum in N-back task, group × time × inter-stimulus interval interaction in dorsolateral PFC, and inferior and superior parietal regions in sustained attention task; (3) –
Siniatchkin et al. (2012)	Nonrandom. contr. (healthy passive contr.)	12 + 12 healthy controls	9.5 and 9.8 years old children with ADHD	Response cost and token-based training; 10-day summer camp	Task fMRI; Go-NoGo paradigm	(1) Increased activation in precuneus, parietal cortex, ACC, and dorsolateral PFC; (2) group × timepoint interaction in dorsal part of ACC; (3) increase in ACC and R dorsolateral PFC correlated with changes in behavioral and clinical variables
Lévesque et al. (2006)	Random. contr. (passive contr.)	15 + 5	10.2 and 10.2 years old unmed. children with ADHD	Neurofeedback training (NFT); 40 sessions over 13 weeks	Task fMRI; Counting Stroop task	(1) Increased activation in R dorsal ACC and L caudate nucleus, not in controls; (2) –; (3) –
Hyun et al. (2016)	Uncontr.	12	10.8 years old children with ADHD	(m) Equine-assisted activity and training; 3 sessions ×70 min over 4 weeks	fMRI; FC	(1) increased FC from cerebellum to R insula, R middle temporal gyrus, L superior temporal gyrus, R precentral gyrus; decreased to L inferior frontal gyrus; (2) –; (3) not for FC
Hoekzema et al. (2010)	Nonrandom. contr. (active contr.)	9 + 10	11 years old children with ADHD	(c) Cognitive training; active controls: social training; 10 days	Task fMRI; paradigms of response inhibition and selective attention	(1) Increased activity after cognitive training in L orbitofrontal, R middle temporal, and dorsolateral PFC, cerebellum, not in controls; (2) group × session interaction; (3) increased activity in cerebellum correlated with improved attention
Hoekzema et al. (2011)	Non-random. contr. (active contr.)	9 + 9	11.3 and 11.2 years old children with ADHD	(c) Cognitive training; active controls: social training; 2 weeks	sMRI	(1) –; (2) increased GM in middle frontal cortex and R inferior-posterior cerebellum compared with controls; (3) GM volume increase in cerebellum correlated with attentional performance improvement
Lee et al. (2017)	Dose contr.	8 + 8 + 7	11.8, 12, and 11.4 years old children with ADHD	(m) Hippotherapy: 1× per week, 2× per week, and 1× per week + neurofeedback; 8 weeks	fMRI	(1) decreased activity in L thalamus in both 1× and 2× groups and increased activity in middle frontal and L precentral cortices in combined group; (2) bigger thalamus change in 1× per week group; (3) –
Maximo et al. (2017)	Random. contr. (passive contr.)	14 + 14	10 and 11 years old children with autism	(c) Visual imagery-based reading intervention; 4 h/day, 10 weeks, 200 h of instruction	Task and rs-fMRI; FC, graph theory	(1) Increased local FC in Reading Network regions; (2) significant compared with controls; (3) changes in L precentral gyrus correlated with improvement in language comprehension
Murdaugh et al. (2015)	Random. contr. (passive contr.)	16 + 15	10.3 and 11 years old children with autism	(c) Visual imagery-based reading intervention; 4 h/day, 5 days/week, 10 weeks	rs-fMRI; FC	(1) Increased FC of Broca's and Wernicke's areas and decreased FC of R inferior frontal gyrus-orbital; (2) changed FC of Broca's and Wernicke's areas; (3) correlated with improvement in reading comprehension
Murdaugh et al. (2016)	Random. contr. (passive contr.)	13 + 13	10.9 and 11 years old children with autism	(c) Visual imagery-based reading intervention; 4 h/day, 5 days/week, 10 weeks	Task fMRI; high- and low-imagery sentence task; FC	(1) Increased activations in high- and low-imagery conditions, not in controls; (2) significant compared with controls; (3) –
Datko et al. (2018)	Uncontr.	10	13.3 years old children with high-functioning autism spectrum disorders	Neurofeedback training (NFT); ≥ 20 h	Task fMRI; imitation and observation task	(1) Increased activation in the R inferior parietal lobule; (2) –; (3) correlated with decreased symptom severity, however in L
Romeo et al. (2018)	Partially random. contr. (passive contr.)	39 + 25	6–9 years old children with reading disability	(c) Intensive summer reading intervention (“Lindamood-Bell”); 6 weeks	sMRI	(1) –; (2) no significant differences in cortical thickness changes between training and control groups; (3) within training group, responders had greater thickening, controls, and non-responders had developmentally typical, non-significant thinning
Iuculano et al. (2015)	Uncontr.	15	7–9 years old children with mathematical learning disabilities	(c) 1:1 math tutoring; 8 weeks	Task fMRI; arithmetic verification—addition task, number identity verification—control task	(1) Decreased functional activation prefrontal, parietal, and ventral temporal-occipital cortices; (2) –; (3) fMRI changes correlated with performance gains
Keller and Just (2009)	Random. contr. (passive contr.)	12 + 25	8–12 years old children who are poor readers	(c) Intensive remedial reading instruction; appx. 100 h over 6 months	DTI	(1) Increased FA with a peak difference in L anterior centrum semiovale, not in controls; (2) group × timepoint interaction for this change; (3) FA increase correlated with improvement in phonological decoding ability
Vander Stappen et al. (2020)	Uncontr.	18	8–12 years old children with dyslexia	(c) Rapid automatized naming (RAN) training	DTI	(1) Not sign. after corr. for multiple comparisons; (2) –; (3) gains in RAN correlated with increased FA in L anterior segment of the arcuate fasciculus (AF)
Huber et al. (2018)	Non-random. contr. (healthy passive contr.)	24 + 19 healthy controls	9.7 and 10 years old children with reading difficulties or dyslexia	(c) Intensive reading intervention; 8 weeks	DTI and tractography	(1) decreased mean diffusivity and increased FA in L AF and L IFL; (2) group × timepoint interaction for this change; (3) mean diffusivity changes correlated with reading skill changes at lag = 0
Horowitz-Kraus and HollS (2015)	Uncontr.	18	9.9 years old children with reading difficulty	(c) Computerized reading acceleration program; 4 weeks	Task fMRI; lexical decision task	(1) Increased FC between L fusiform gyrus and R anterior cingulate cortex; (2) –; (3) the change in FC correlated with increased behavioral scores
Gebauer et al. (2012)	Random. contr. (passive contr.)	10 + 9	11.5 and 12.1 years old children with poor spelling abilities	(c) Computer-aided morpheme-based spelling training; 5 weeks	DTI	(1) –; (2) greater decrease of mean diffusivity in R superior corona radiata compared with waiting controls; (3) –
Sterling et al. (2013)	Contr. within-subject	10	3 years old children with congenital hemiparesis	(m) Constraint-induced movement therapy	sMRI	(1) Increased GM volume in sensorimotor cortex contralateral to more-affected arm, not during control period; (2) –; (3) not significant (trends)
Kwon et al. (2014)	Uncontr.	5	3 years old before excl. children with unilateral cerebral palsy	(m) Constraint-induced movement therapy; 4 weeks, 120 h of constraint	DTI and tractography	(1) Increased, FA, fiber number in the affected corticospinal tract and decreased asymmetry (no stats.); (2) –; (3) –
Lee et al. (2014)	Uncontr.	2	6 and 9 years old children with spastic hemiplegic cerebral palsy	(m) Intensive hand strengthening training; 60 min/day, 3 times/week, for 10 weeks	Task fMRI; motor task	(1) Decreased activation/location change in both children (no stats); (2) –; (3) –
Soh et al. (2015)	Partially random. contr. (passive contr.)	20 + 18 + 19 healthy controls	9.5, 9.9, and 10 years old children with fetal alcohol spectrum disorder	(c) Alert program for self-regulation; 12 weeks	sMRI	(1) Increased GM in L middle frontal gyrus, R frontal pole, and R ACC, not in controls or healthy controls (uncorrected); (2) –; (3) increased GM volumes of frontal gyri correlated with behavioral improvements
Everts et al. (2017)	Random. contr. (passive contr. + 2 memory training types)	23 + 23 + 23 controls	10, 10.7, and 9.8 years old very preterm born or very low birth weight children	(c) Training group 1: memory strategy training; training group 2: intensive working memory practice	Task fMRI; visual working memory (Dot Location) task	(1) Decrease of intensity of brain activation in frontal brain region in training group 1 and in R frontal and parietal regions in training group 2, none in controls; (2) –; (3) –
Nash et al. (2017)	Random. contr. (passive contr.)	10 + 11	10.3 and 10.4 years old children with fetal alcohol spectrum disorder	(c) Alert® program for self-regulation; 12 1.5 h sessions over ~14 weeks	Task fMRI; Go-NoGo paradigm	(1) –; (2) group × timepoint interaction in R superior frontal gyrus, L middle frontal, L inferior parietal lobule; (3) –
Phillips et al. (2007)	Uncontr.	3	10.5 years old before exclusion ambulatory children with spastic cerebral palsy	(m) Intensive body-weight-supported treadmill training (BWSTT); 12 sessions over 2 weeks	Task fMRI; ankle dorsiflexion and finger tapping	(1) Increased cortical activation during ankle and hand tasks in all 3 children (no stats.); (2) –; (3) –
Kesler et al. (2011)	Uncontr.	11	10.9 years old before excl. females with Turner syndrome (TS)	(c) Adaptive computerized math training; 20 min × 5 days/week for 6 weeks	Task fMRI; math task	(1) Increased parietal activation and decreased frontal-striatal and mesial temporal activation; (2) –: (3) –
Weinstein et al. (2015)	Uncontr.	12	11 years old children with hemiparesis	(m) Hand-arm bimanual intensive therapy (HABIT); 60 h over 2 weeks	Task fMRI; motor task; DTI	(1) Increased activation in the affected hemisphere, increased lateralization index, no DTI changes; (2) –; (3) –
Cope et al. (2010)	Uncontr.	7	11 years old before excl. children with hemiplegia	(m) Constraint-induced movement therapy; 2 weeks, total of 10 treatment days, 40 h of therapy	Task fMRI; hand tapping	(1) Increased activation in 3 out of 7 subjects, no stats. reported; (2) –; (3) –
Riggs et al. (2017)	Quasi-random. contr. (crossover passive contr.)	16 + 12	11.2 and 12 years old children treated with radiation for brain tumors	(m) Exercise training; 12 weeks	sMRI; DTI	(1) –; (2) increased FA in corpus callosum, cingulum, superior longitudinal fasciculi, R corticospinal tract, and inferior frontal occipital fasciculus, increased hippocampal volume; (3) increased FA correlated with decreased reaction time
Szulc-Lerch et al. (2018)	Quasi-random. contr. (crossover passive contr.)	16 + 12	11.2 and 12 years old children treated with radiation for brain tumors	(m) Exercise training; 12 weeks	sMRI	(1) –; (2) increased cortical thickness and WM volume primarily in motor/sensory cortex; (3) not significant (trend) correlation with changes in accuracy and reaction time
Zou et al. (2012)	Effectively uncontr.	14 before excl.	12 years old before excl. children who are cancer survivors	(c) Cognitive remediation program; 20 sessions over 4–5 months	Task fMRI; Conner's continuous performance task	(1) Increased activation in cerebellum, fusiform, L inferior frontal area and insula, deactivation in precuneus, and postcentral gyrus; (2) –; (3) –
Conklin et al. (2015)	Uncontr.	34	12.2 years old survivors of childhood acute lymphoblastic leukemia or brain tumor	(c) Computerized cognitive intervention (Cogmed); 25 sessions over 5–9 weeks	Task fMRI; grid-based spatial working memory task	(1) Decreased activation of L lateral prefrontal, L cingulate, and bilateral medial frontal areas; (2) –; (3) no correlation
Yuan et al. (2017)	Nonrandom. contr. (healthy passive contr.)	10 + 11 healthy controls	13.7 years old before excl., 13.4 years old children with TBI	(c) Attention Intervention and Management (AIM) program; 10 weeks	DTI tractography; SC, graph theory	(1) Decreased small-worldness and normalized clustering coefficient; (2) group × timepoint interaction for these 2 metrics; (3) no correlations for those 2 metrics but for mean local efficiency and path length
Juenger et al. (2013)	Uncontr.	16	16.4 years old children and young adults with congenital hemiparesis	(m) Constraint-induced movement therapy (CIMT); 12 days of individual (2 h/day) and group training (8 h/day)	Task fMRI; unimanual (active or passive) hand movement	(1) Decreased primary motor activation in ipsilateral group but increased motor and somatosensory activation in contralateral group; (2) –; (3) –
Augustijn et al. (2019)	Non-random. contr. (healthy passive contr.)	19 + 24 controls	7–11 years old children with obesity	Multidisciplinary treatment: diet restriction, CBT, and physical activity; 11 months	sMRI, DTI, magnetization transfer	(1) Significant increase in total and cerebellar GM volume, not in controls; (2) –; (3) only weak to moderate (nonsign.) correlations between structural brain alterations and behavioral improvements
Davis et al. (2011)	Random. contr. (passive contr.)	10 + 9 controls	7–11 years old overweight children	(m) Aerobic exercise; 14 weeks, 20–40 min daily	Task fMRI; executive function (antisaccade) task	(1) –; (2) increased prefrontal cortex activity and decreased activity in posterior parietal cortex compared with controls; (3) –

An expanded version of the table is available on the Open Science Framework (OSF) website: https://osf.io/4zckm/.

*ACC, anterior cingulate cortex; ADHD, attention-deficit/hyperactivity disorder; appx., approximately; CBT, cognitive-behavioral therapy; (c), cognitive training; DTI, diffusion tensor imaging; excl., exclusion; FA, fractional anisotropy; FC, functional connectivity; fMRI, functional MRI; GM, gray matter; IPC, inferior parietal cortex; L, left; MDD, major depressive disorder; (m), motoric training; MRSI, spectroscopic imaging; (n), normal population; OCD, obsessive-compulsive disorder; OFC, orbitofrontal cortex; PFC, prefrontal cortex; PTSD, posttraumatic stress disorder; R, right; random., randomized; rs-fMRI, resting-state fMRI; (s), special population; SC, structural connectivity; sMRI, structural MRI; TBI, traumatic brain injury; unmed., unmedicated; WM, white matter*.

*Sample Size*. The number of subjects who participated in the training of interest and underwent neuroimaging was below 30 in most studies (63 out of 71 studies, 89%).

*Population*. We generally categorized study participants younger than 13 years old as children and those who were 13 years old or older as adolescents, since the pubertal status was not always available. The majority of the studies (*N* = 48, 67.6%) were on children and only two studies involved adolescents and children, limiting the basis for comparisons across age groups. Most of the studies (*N* = 55; 77.5%) used a special (mostly clinical) population. For instance, there were 9 studies involving ADHD, four on autism, and seven on dyslexia or dyscalculia, while 9 dealt with affective disorders and challenges.

*Training Type*. Training types ranged from math and piano lessons, to cognitive-behavioral therapy (CBT), to reading training, memory training, exercise, meditation, hippotherapy, and neurofeedback. Most prevalent training type was cognitive (*N* = 46; 64.8%), whereas 12 studies (16.9%) involved mainly motoric and 18.3% mixed training type. The type of training was distributed depending on population. Among the studies conducted by sampling from the normal population, all but one (mixed) used cognitive training, whereas there were 31 studies with cognitive training and 12 each with mixed and motoric training conducted with a special population [$\chi_{\left(2\right)}^{2}$ = 7.81, *p* = 0.02]. Motoric training was only present among studies involving children (*N* = 12), whereas there were six mixed training studies each with children and with adolescents, as well as 30 cognitive training studies with children and 15 with adolescents [$\chi_{\left(2\right)}^{2}$ = 7.6, *p* = 0.022]. Participants were younger in studies with motor interventions (*M* = 9.4 years, SD = 3.94 years) than in studies with mixed interventions [*M* = 12.5 years, SD = 3.1 years, *t*_(23)_ = 2.22, *p* = 0.037], whereas studies with cognitive interventions were in between (*M* = 11.1 years, SD = 3.1) and did not differ in participant age either from the mixed intervention studies [*t*_(57)_ = 1.45, *p* = 0.153] or from the motor studies [*t*_(56)_ = 1.62, *p* = 0.11].

*Type of MRI*. In 70.4% (*N* = 50) of the studies, functional MRI was involved. Specifically, 58% of the studies were task-based fMRI studies utilizing a variety of tasks. Other studies employed resting-state fMRI, structural MRI, DTI, and MRSI. Studies involving functional imaging were on average conducted with older children (average lower age bound of study: *M* = 11.7 years, SD = 2.8 years), whereas the lower age bound of the sample was on average lower in studies only involving structural measures [*M* = 9.7 years, SD = 4 years, *t*_(69)_ = 2.31, *p* = 0.024].

Hypothesis H1, stating that there are differences in the MRI-derived neural metrics before and after the training in children and adolescents, was explicitly tested in 56 (79%) of the studies. The results suggest that there are differences in the MRI-derived neural metrics before and after the training in children and adolescents. Only six studies (11%) showed no significant changes in MRI measures in youth with training (Jolles et al., 2012, 2013, 2016b; Catharine et al., 2019; Karoly et al., 2019; Vander Stappen et al., 2020), whereas the remaining 50 studies (89%) showed some changes. Additionally, four studies involving very small numbers of subjects did not report inferential statistical results (Phillips et al., 2007; Cope et al., 2010; Kwon et al., 2014; Lee et al., 2014). Thus, 46 studies out of 56 studies with statistical analysis (82%) showed statistically significant neural changes with training.

Hypothesis H2, stating that there are differences in changes of the MRI-derived neural metrics between the training group and control group in children and adolescents, was explicitly tested in 34 (48%) of the studies. The results suggest that there are differences in changes of the MRI-derived neural metrics between the training group and control group in children and adolescents. The 34 studies utilized some type of a control condition discussed in the “INTRODUCTION” (Table 2), including within-subject and non-randomized designs. One study used a different type of control condition not discussed above: They compared GM volume changes in executive functioning regions compared with control regions in the same subjects (Catharine et al., 2019). Twenty-one studies used a randomized controlled design (30% of all reviewed studies), with 10 of them using an active control group (14% of all reviewed studies). Significant differences compared with the control condition were found in 30 (88%) of the studies that tested H2. Overall, when combining the H1 and H2 results, some neural changes with training (with or without control condition) were reported in 62 (87%) of the 71 studies.

Hypothesis H3, stating that the improvement in performance in the training group (or among responders) correlates with the MRI-derived neural changes in children and adolescents, was explicitly tested in 45 (63%) of the studies. The results suggest that the improvement in performance in the training group (or among responders) correlates with the MRI-derived neural changes in children and adolescents. Specifically, 36 of the 45 studies (80% of the studies that tested H3; 51% of all studies) found significant correlations.

The directionality of the observed changes in the MRI metrics with training strongly depended on the type of MRI modality, specific brain region(s), and nature of training (Table 3, last column). The effect size was only reported in seven studies. In the first study, participants in CBT, relative to controls, showed a significant activation increase in right ventrolateral prefrontal cortex in response to angry faces, *t*_(15)_ = 3.22, *p* < 0.01, effect size *d* = 1.28, cluster size = 29 voxels at *p* < 0.01 (Maslowsky et al., 2010). However, the authors warn that this large effect size (Cohen, 1988) should be interpreted with caution, since the use of a cluster derived from activation may inflate effect size estimate (Poldrack and Mumford, 2009). Three other fMRI activation studies reported effect sizes of Cohen's *d* 0.36–0.48 (Iuculano et al., 2015), 1.04 (Rosenberg-Lee et al., 2018), and 0.91–2.21 (Karoly et al., 2019). It needs to be noted that while Rosenberg-Lee et al. (2018) reported increases, Karoly et al. (2019) and Iuculano et al. (2015) reported decreases in brain activation with training, which can be explained by the different nature of training, brain regions, and fMRI tasks [e.g., arithmetic task used by Rosenberg-Lee et al. (2018) vs. visual cannabis cue-reactivity task used by Karoly et al. (2019)]. In the fifth study, Huber et al. (2018) reported effect sizes of Cohen's *d* = 0.75 and 0.66 for DTI changes after reading training. In the sixth study, the authors used connectomics analysis, in which only a limited number of global (summary) network metrics were derived from whole-brain diffusion MRI images (Yuan et al., 2017). Participants with traumatic brain injury underwent attention training and were compared with passive controls. The normalized clustering coefficient and small-worldness displayed significant group × timepoint interactions, both with large effect sizes: −1.1 and −1.4, respectively. Finally, in the seventh study, an effect size of Cohen's *d* = 0.47 was reported for gray matter volume decreases in the left posterior insula in adolescents with meditation training (Yuan et al., 2020). Interestingly, the authors report that the observed VBM changes with meditation training in the studied sample of adolescents were opposite in directionality compared with the previously published VBM literature in adults with meditation training (Yuan et al., 2020).

## Discussion

This systematic review of MRI studies of training-induced neuroplasticity in children and adolescents has been conducted in order to investigate, whether there are measurable neural effects of training in children and adolescents that can be captured by MRI. We hypothesized that: (1) there would be differences in the MRI-derived neural metrics before and after the training; (2) there would be differences in changes of the MRI-derived neural metrics between the training group and control group; and (3) the improvement in performance in the training group (or among responders) would correlate with the MRI-derived neural changes.

A surprisingly large number of studies met the inclusion criteria: a total of 71 articles were included in the review. Significant changes in brain activation, structure, microstructure, and structural and functional connectivity were reported with different types of trainings in 62 studies (87% of studies), thus supporting the hypothesized neural changes with training. This is a percentage on the higher end of what is generally observed in hypothesis testing studies in the fields of psychology, clinical medicine, and neuroscience and behavior (Fanelli, 2010). Both the effect size and directionality strongly depended on the type of MRI modality, specific brain regions, and nature of training. The effect size is rarely reported in neuroimaging studies, where a large number of non-independent data points are frequently collected and then the same hypothesis test is run on tens of thousands of voxels. Among the included studies, the effect size was only reported in seven studies, ranging from 0.36 to 2.2 (medium—large effect sizes, Cohen, 1988).

The reason for the predominantly positive results can be the presence of reporting biases: publication bias (e.g., selective reporting of complete studies; Dickersin, 2005) as well as the “outcome reporting bias” (*p*-hacking) within individual studies (Chan et al., 2004). The latter bias is especially likely in the case of MRI-based analyses of training-induced neuroplasticity due to the multitude of brain regions, metrics, and preprocessing steps that can be potentially employed. Poldrack et al. (2017) recently discussed, for example, that in the fMRI analysis, one can apply 6,912 analysis workflows to a single data set. Although not all combinations of parameters are plausible and most studies share very similar workflow, the hidden flexibility in MRI data analysis theoretically “allows presenting anything as significant” (Simmons et al., 2011; p. 1359). Nevertheless, the authors suggest that the best solution is to allow for this flexibility but to require that researchers: (1) preregister the methods and analysis plans, for example, using the OSF and (2) clearly indicate which analyses are exploratory and ideally validate exploratory results, for example, by using a separate validation data set. Finally, a potential means to detect the outcome reporting bias among the studies is to apply *p*-curve analysis (Simonsohn et al., 2014), which would need to be adapted to neuroimaging, where the hypothesis is often tested for tens of thousands of non-independent voxels in the brain followed by various methods to correct for multiple comparisons.

Unfortunately, the majority of included studies were designed in such a way that only a positive outcome would be informative, thus contributing to the publication bias. That is, they were largely underpowered and therefore a null result would not give any useful information: 89% of the included studied had fewer than 30 subjects who participated in the training of interest and underwent neuroimaging. This does not mean that “null results should not be published, only that studies should be designed so that null results are worth publishing” (Green et al., 2014; p.769). It is surprising that randomized-controlled trials (RCTs) are often accompanied only by sample size calculations to detect clinical improvements, whereas the corresponding calculations for the changes in neuroimaging measures are lacking, despite the high costs of neuroimaging (Reid et al., 2017).

Another reason for the largely positive findings can be the absence of a proper control group that would allow to rule out developmental changes or regression toward the mean in clinical populations. Only 21 of the included studies used a randomized controlled design (30%), with only ten of them using an active control group. Overall, some type of control (including within-subject and non-randomized designs) was used in 34 (48%) of the studies. Thus, our Hypothesis 2, stating that there are differences in changes of MRI-derived metrics between the training group and control group, was tested by 34 studies. It needs to be noted that some of the studies had a passive control group consisting of a *different* population than the main studied one, for example, typically developing children instead of children with dyslexia (Huber et al., 2018). This limits our ability to make statements about differences in brain changes with vs. without training in the population of interest. We did not consider a study to have a control condition when a different population underwent the *same* type of training as the population of interest (e.g., Bauer et al., 2019).

Finally, the positive findings can reflect the actual impressive capacity of the developing brain to demonstrate measurable plasticity with training. Organization of human cerebral cortex is significantly less genetically heritable than that of non-human primates, which implies a higher degree of neuroplasticity and stronger environmental influence on brain development in our species (Gómez-Robles et al., 2015). This relative lack of genetic influence is most noticeable in association areas of the brain and is likely reflected in microstructural adaptations in neural circuitry. An important implication of this heightened neuroplasticity is that “the development of neural circuits that underlie behavior is shaped by the environmental, social, and cultural context more intensively in humans than in other primate species, thus providing an anatomical basis for behavioral and cognitive evolution” (Gómez-Robles et al., 2015; p. 14799).

Most studies that looked at the correlation of brain changes and behavior reported a significant correlation for at least one of the brain region's property with at least one behavioral metric (36 studies or 80% of the 45 studies that tested H3). Null results obtained in 9 studies may reflect the underpowered design or the fact that it is not always clear, which behavioral measure is supposed to correlate with the neural change. Finally, it can be a timing issue and the lack of correlation may be due to differences in temporal dynamics of neural and behavioral changes. As discussed in “**Training-Induced Biological Changes Measured With MRI**,” changes in performance that take place with training may have different timing compared with changes in various neural measures (Kelly and Castellanos, 2014). It may be in part due to the complex reciprocal causality between brain and behavior/performance. While the title of this paper implies that the causality arrow points from behavior (training) to neural remodeling, we also expect the reverse causality to take place: we hypothesize performance improvements caused (mediated) by this neural remodeling. One of the insights from research on Alzheimer's, Parkinson's, Huntington's, and many other brain disorders is that “behavior is the last thing to change” (Insel, 2013). It is therefore plausible to expect situations in which neural remodeling can be detected using MRI before stable performance improvement is observed. In general, it has been argued that neuromarkers often provide better predictions (neuroprognosis) of future (1) education, learning, and performance in children and adults; (2) criminality; (3) health-related behaviors; and (4) responses to pharmacological or behavioral treatments, than traditional behavioral measures (Gabrieli et al., 2015). The exact timing of neural and behavioral changes with different types of training still remains a topic for future research.

### Recommendations for Future Studies

Based on the results of this systematic review, the following recommendations for future studies were derived:

- Having appropriate study design, ideally with randomization and active controls (Table 2);
- Having transparency. As discussed above, to increase transparency one can: (1) preregister the methods and analysis plans, for example, using the OSF; (2) clearly indicate which analyses are exploratory. Finally, data and code sharing can enable direct replication and thus provide maximum transparency;
- Taking into account behavioral and physiological changes (e.g., cardiac pulsation) that might affect MRI measures without reflecting underlying neuroplasticity (Table 1);
- Taking into account the complex developmental changes in MRI-derived metrics observed with age when interpreting brain-behavior correlates (Table 1). As discussed above, opposite correlations with performance can be observed in children compared with adults, e.g., in one study, thinning of the cortex was associated with higher intelligence at younger age, however, this relationship was reversed in young adults, where higher IQ correlated positively with increase in cortical thickness (Schnack et al., 2015). Similarly, one of the reviewed training studies (Yuan et al., 2020) reported that the observed VBM changes with meditation training in the studied sample of adolescents were opposite in directionality compared with the previously published VBM literature in adults with meditation training, which may be due to developmental synaptic pruning effects (Giedd et al., 1999);
- Examining long-term effects of the training by conducting additional measurements months or years after the training (e.g., as it was done in Sotnikova et al., 2012);
- Taking into account general methodological considerations that apply to all research of training, such as the lack of transfer effects known as the “curse of specificity” (Green et al., 2014). The curse of specificity refers to the situation in which individuals improve on a task with training but when a new (even very similar) task is given, no benefit of the training is observed (Green et al., 2014). This is especially the case for the laboratory based as opposed to ecological interventions (Bryck and Fisher, 2012). Another of such general methodological issues is the expectation effect (Green et al., 2014). An ethically controversial approach would be to have a control group merely for the expectation effect (i.e., telling the participants in this control group that they are in the “experimental” condition where behavioral improvements are expected). Finally, an often unresolved methodological question in training research is the number of administered tests (in case of MRI studies, both for behavioral and MRI measures). Having a large number of tests has both strong advantages (e.g., the possibility of making inferences for various measures) as well as disadvantages (e.g., cognitive fatigue or reductions in statistical power due to correction for multiple comparisons) and needs to be justified. The order of the tests should ideally vary randomly across participants to prevent one or several of the tests from carrying a disproportionate amount of order effects such as fatigue and compliance.

### Future of Neuroplasticity Research

It can be expected that neuroimaging studies of neuroplasticity will continue gaining importance. In general, there are two large areas of applying the gained knowledge on neuroplasticity: clinical and educational.

- *Clinical Applications*. The majority of training studies in medical field have traditionally been concerned exclusively with the efficacy of the training, rather than *why* the training is effective (i.e., the mechanism). Recently, the National Institutes of Health (NIH) directed their focus on funding of “mechanistic” studies that would demonstrate that the hypothesized mechanism of action is indeed triggered by the intervention, before conducting a full-size RCT. Neuroimaging plays a central role in this type of studies. Future studies may also shed light on the “dark side” of brain plasticity. Neuroplasticity, the brain's response to environmental experience, resembles a double-edged sword: on one hand, it potentiates growth or recovery when an individual is exposed to normative, benign, or therapeutic environments (Sonuga-Barke, 2017). On the other hand, in an individual exposed to adversity (e.g., childhood maltreatment), the neurocognitive changes in response to the adversity can be seen as beneficial/functional and have adaptive value within that particular context but can mediate mental disorder development in the long-term (McCrory et al., 2017; Sonuga-Barke, 2017). Better understanding of this type of plasticity can have tremendous clinical significance.
- *Educational Applications*. Training of a specific brain function may require a certain stage of brain development. Curriculum development in the educational system will greatly benefit from the knowledge of such brain development milestones, as well as of sensitive periods (developmental periods during which experience has the most long-lasting effects). The proper foundation for brain-based learning is not yet available to educators, but research is being conducted and neuroimaging technology will keep playing an important role in advancing our knowledge of the developing brain.

In either content domain, future neuroplasticity research can benefit from measurements and models that allow to capture intervention-based change in the brain as well as other brain changes over time. For instance, Kievit et al. (2018) suggest applying latent change score models in longitudinal samples. In part, they can be of use even when there are only two measurement occasions.

### Limitations of the Review

There are several limitations to this review. First, because a meta-analysis was not appropriate (see the earlier remarks in this respect) and only few of the studies reported effect size, only very general findings are reported (Table 3). Heterogeneity of MRI modalities, post-processing methods, training types, and brain regions analyzed additionally complicated comparability of results. Both increases and decreases in MRI-derived measures were observed (variable effect direction; Table 3).

A second limitation was our focus only on mechanistic, not prognostic, imaging biomarkers. Prognostic role of imaging may play a critical role in treatment planning of psychiatric and neurologic disorders in the future.

Finally, this review focused on training studies in the developing brain only and did not include comparison between pediatric and adult brain studies of neuroplasticity. To better probe developmental aspects of neuroplasticity, one interesting hypothesis for future reviews would be that the effect size in the young brain is larger than in the adult brain. In one study included in this review, similar reductions in subsequent-memory effects in task-positive brain regions were found after strategy training across age groups: in children, younger adults, and older adults (Brehmer et al., 2016). Another study included in this review directly compared effects of working memory training in children and adults, although inclusion of children was a pilot and both groups were uncontrolled (Jolles et al., 2013). The results did not show practice effects on functional connectivity in children, only in adults. This preliminary finding argues against the hypothesis of stronger neuroplasticity in a younger brain. One possible reason for the lack of the effect in children is the immature structure of their brains. As an alternative explanation, the authors suggest that children already experience working memory practice in school and therefore extra training has less impact on functional connectivity (Jolles et al., 2013). Future studies might focus on comparing children and adolescents (rather than children and adults) as the onset of puberty may affect sensitive phases for cognitive and brain development (see Laube et al., 2020, for a review). For instance, there are recent discussions on whether plasticity for higher order cognitive functioning is higher or lower during adolescence compared with childhood (e.g. Fuhrmann et al., 2015; Piekarski et al., 2017; Laube et al., 2020).

In spite of these limitations, this review provides a comprehensive summary of the published MRI-based findings in youth with various types of mental and behavioral training. On the one hand, the results suggest that there is substantial evidence for training-induced neuroplasticity with different types of training captured with different MRI measures in children and adolescents. The numerous studies contributing to this field are highly diverse with respect to training and population used as well as design and measures. At the same time, our review makes explicit that there are frequent methodological limitations (such as small sample sizes, lack of an adequate control group). The presented overview of the work on training-induced neuroplasticity in adolescents and children offers a possibility to critically evaluate the strength of the accumulated evidence and to provide recommendations for future research.

## Author Contributions

OT and RG formulated the research question. OT conducted literature search and data extraction, and RG supervised this process. OT and RG wrote and revised the manuscript. All authors contributed to the article and approved the submitted version.

## Conflict of Interest

The authors declare that the research was conducted in the absence of any commercial or financial relationships that could be construed as a potential conflict of interest.
